# Development and Characterization of an Amorphous Solid Dispersion of Furosemide in the Form of a Sublingual Bioadhesive Film to Enhance Bioavailability

**DOI:** 10.3390/pharmaceutics9030022

**Published:** 2017-06-24

**Authors:** Viviana De Caro, Alessia Ajovalasit, Flavia Maria Sutera, Denise Murgia, Maria Antonietta Sabatino, Clelia Dispenza

**Affiliations:** 1 Dipartimento di Scienze e Tecnologie Biologiche Chimiche e Farmaceutiche (STEBICEF), Università degli Studi di Palermo, Palermo, PA 90123, Italy; denisemurgia@libero.it; 2 Dipartimento dell'Innovazione Industriale e Digitale, Ingegneria Chimica, Gestionale, Informatica, Meccanica, Università degli Studi di Palermo, Palermo, PA 90128, Italy; alessia.ajovalasit@unipa.it (A.A.); mariaantonietta.sabatino@unipa.it (M.A.S.); clelia.dispenza@unipa.it (C.D.); 3 SiSaf Ltd, Innovation Centre, Northern Ireland Science Park, Queen’s Island, Belfast BT3 9DT, UK; flaviam.sutera@gmail.com; 4 Consiglio Nazionale delle Ricerche, Istituto di Biofisica–UOP Palermo, Palermo, PA 90146, Italy

**Keywords:** mucoadhesive film, sublingual absorption, amorphous solid dispersion, furosemide bioavailability, transmucosal delivery

## Abstract

Administered by an oral route, Furosemide (FUR), a diuretic used in several edematous states and hypertension, presents bioavailability problems, reported as a consequence of an erratic gastrointestinal absorption due to various existing polymorphic forms and low and pH-dependent solubility. A mucoadhesive sublingual fast-dissolving FUR based film has been developed and evaluated in order to optimize the bioavailability of FUR by increasing solubility and guaranteeing a good dissolution reproducibility. The Differential Scanning Calorimetry (DSC) analyses confirmed that the film prepared using the solvent casting method entrapped FUR in the amorphous state. As a solid dispersion, FUR increases its solubility up to 28.36 mg/mL. Drug content, thickness, and weight uniformity of film were also evaluated. The measured Young’s Modulus, yield strength, and relative elongation of break percentage (EB%) allowed for the classification of the drug-loaded film as an elastomer. Mucoadhesive strength tests showed that the force to detach film from mucosa grew exponentially with increasing contact time up to 7667 N/m^2^. FUR was quickly discharged from the film following a trend well fitted with the Weibull kinetic model. When applied on sublingual mucosa, the new formulation produced a massive drug flux in the systemic compartment. Overall, the proposed sublingual film enhances drug solubility and absorption, allowing for the prediction of a rapid onset of action and reproducible bioavailability in its clinical application.

## 1. Introduction

Hypertensive emergencies and hypertensive urgencies are commonly encountered by a wide variety of clinicians. Prompt recognition, evaluation, and appropriate treatment of these conditions are crucial to prevent permanent organ damage [1].

Furosemide (FUR) is a loop diuretic used orally in the treatment of edematous states associated with cardiac, renal, and hepatic failures and in the treatment of hypertension [2]. The usual dosage is 40–120 mg/day. For the treatment of mild cases of edema, doses as low as 20 mg can be effective, whereas for severe cases, doses as high as 600 mg/day may be required [3].

Data on solubility, oral absorption, and permeability are sufficiently exhaustive to classify FUR into Class IV of the Biopharmaceutics Classification System. Due to the carboxyl and sulfonamidic groups in the structure, with pKa of 3.8 and 9.6, respectively [4], FUR shows a very low solubility in water that increases as a function of pH from 0.01 mg/mL at pH 2 to 1.9 mg/mL at pH 7.4 [5], determining a higher absorption in the gastric rather than intestinal tracts. A erratic bioavailability of about 37–51% and large inter- and intra-subject variabilities in rate and the extent of absorption were also reported [6]. Absorption following oral administration is influenced by the dosage form, underlying disease processes, and by the presence of food. The intra-subject variability was thought to be mainly dependent on the absorption process, since repeated intravenous (i.v.) doses showed only marginal variability [7].

The bioavailability problems, reported as consequences of variable and erratic gastrointestinal absorption, are probably due to the low and pH-dependent solubility together with various existing polymorphic forms of FUR. [8]. Indeed, FUR presents different polymorphic forms: four are true polymorphs (I, II, III, IV), two are solvates (IV-DMSO and V-dioxane) and one is an amorphous form [9,10]. It is well known that different polymorphic forms of an active administered in the oral or topical form can modify many properties like solubility, stability, color, compressibility, flowability, and workability and as a consequence can cause differences in bioavailability, toxicological safety, clinical effectiveness, and productive efficiency [11].

Different approaches have been proposed to enhance FUR absorption and bioavailability such as co-crystallisation [12,13], solid dispersion [14], microemulsification [15], and supramolecular complexes formation [16].

Oral mucosal drug delivery is an alternative and promising method of systemic delivery which offers several advantages [17,18]. The oral mucosa is highly vascularized; for this reason, drugs that are absorbed through it directly enter the systemic circulation, bypassing the gastrointestinal tract and first-pass metabolism in the liver. [19]. When sublingual mucosa administration is chosen, a rapid onset of action results via a more comfortable and convenient delivery route than the intravenous route. [20]. Additional advantages include an improved bioavailability for certain drugs and an easy access to the absorption sites so that the delivery system can be applied and removed easily [21,22]. Fast dissolving oral film, in particular when placed in the oral cavity, rapidly disintegrates and dissolves to release the medication without chewing and intake of water [23,24]. It gives quick absorption and bioavailability comparable to intravenous administration.

Bioadhesive sublingual formulations [25], such as tablets [26], patches, and films, have been developed using mucoadhesive polymers that can establish a strong adhesive contact with the mucosa, allowing for an increase in residence time of the delivery system and optimizing drug bioavailability [19,27]. The release kinetics of a given drug from a polymeric matrix could be governed predominantly by the polymer morphology and excipients present in the system [28].

Furthermore, a comparative study on pharmacokinetics and pharmacodynamics of FUR after administration of the same tablets for both sublingual and oral routes, using the i.v. route as control, has been reported. The authors concluded that the insignificant differences observed between oral and sublingual routes were due to the slow disintegration of the oral tablet that produces a variable dose fraction swallowed in the sublingual environment and that a dosage form suitably formulated to be applied on the sublingual mucosa would improve the bioavailability of FUR [29].

As a consequence, the aim of this work is the design of an adequate new formulation capable of improving the pharmacokinetics of FUR when administered sublingually.

In this study, a mucoadhesive sublingual fast-dissolving film loaded with amorphous FUR has been developed. The formulation was designed in order to optimize the bioavailability of FUR by increasing solubility and guarantying good dissolution profile reproducibility and massive transmucosal absorption.

## 2. Experimental

### 2.1. Materials

FUR, Ph. Eur. grade, was purchased from Galeno srl (Carmignano, PO, Italy). Methacrylic acid-methyl methacrylate copolymer (Eudragit^®^ L-100) was kindly supplied by Rofarma (Milan, Italy). Polyvinylpyrrolidone K-90 (PVP K-90), Sorbitol, Agar, Triethanolamine (TEA), and NaOH were purchased from Sigma-Aldrich (Milano, Italy). Propylene glycol was purchased from Farmalabor (Canosa di Puglia, Italy).

Buffer pH 6.8 solution simulating saliva was prepared using NaCl, KCl, KSCN, KH_2_PO_4_, Urea, Na_2_SO_4_ 10H_2_O, NH_4_Cl, CaCl_2_·2H_2_O, and NaHCO_3_ in distilled water according to Gal et al. [30]. A total of 6 mM phosphate buffered saline (PBS), Ca^2+^, and Mg^2+^ free solution, simulating pH 7.4 plasma, was prepared by dissolving KH_2_PO_4_, anhydrous Na_2_HPO_4_, and NaCl in distilled water. A 0.9% saline solution was prepared by dissolving NaCl in distilled water.

All chemicals and solvents of analytical grade were purchased from VWR International (Leuven, Belgium) and used without further purification. Porcine mucosae were kindly supplied by the Municipal Slaughterhouse of Villabate (Palermo, Italy).

### 2.2. Methods

#### 2.2.1. Preparation of Mucoadhesive Film

TEA (450 mg) and FUR (600 mg) were added to an aqueous solution (10 mL) of propylene glycol (150 mg), Sorbitol (80 mg), and PVP-K90 (40 mg), and the resulting solution was stirred until complete dissolution. Then, Eudragit L100^®^ (1 g) and NaOH 1 M (5 mL) were added to the solution until a fluid, transparent, and homogeneous viscous solution was formed. The mixture was poured into a silicon mold having an area of 20.25 cm^2^ and dried in an oven at 40 °C for 48 h. The so formed films (plain patches) were then left to equilibrate at room temperature and humidity for 24 h, checked for any imperfections or air bubbles, and cut by a biopsy punch into disks 8.0 mm in diameter (area 0.5 cm^2^). The samples were packed in polyethylene bags, heat-sealed, and stored at room temperature in a glass container to maintain the integrity and elasticity of the films.

#### 2.2.2. Film Weight, Thickness, and Drug Load Uniformity

Five disks from each batch were randomly cut and weighed. The diameter and thickness were measured with a vernier caliper and an analog micrometer (Mitutoyo Italiana SRL, Milano, Italy), respectively.

Drug content was estimated by dissolving a randomly selected disk, by sonication, into a 200 mL flask filled to volume by distilled water. The amount of FUR released from the film was measured spectrophotometrically (UV/Vis Shimadzu model 1700 instrument, Japan) at λ_max_ = 331 nm, (linearity range 0.005–0.1 mg/mL, E_1%_water = 0.158, corr. coeff. 0.999).

The uniformity of batches was evaluated by calculating the averages and standard deviations for all the considered parameters.

#### 2.2.3. Differential Scanning Calorimetry (DSC)

Differential Calorimetric Analysis was performed on the patches and on their individual components (sample weight was approx. 8 mg) by a Perkin Elmer Jade calorimeter with a temperature ramp from 30 °C to about 250 °C at 10 °C/min, under nitrogen flow.

#### 2.2.4. Attenuated Total Reflectance (ATR)

ATR-FTIR spectra were recorded on a Fourier Transform Infrared Spectrometer (FTIR) (Spectrum Two FTIR spectrometer, Perkin Elmer) equipped with an ATR unit plug-and-play with a diamond crystal for surface analysis. Spectra were obtained by accumulation of 32 scans between 4000 and 450 cm^−1^ at 4.0 cm^−1^ resolution and rationed to the appropriate background spectra.

#### 2.2.5. Mechanical Tests

Mechanical properties of films were evaluated using an Instron Universal Testing instrument (model 3365 equipped with 8500 digital control, Instron, Norwood, MA, USA) with a 1 kg load cell. A specimen with dimensions of 10 × 60 × (0.9 ± 0.12) mm was held between two grips covered by a felt pad to prevent slippage and positioned at a distance of 2 cm. A tensile test was performed at a rate of 100 mm/min. Tensile strength and elongation at break were measured. Reported data are averaged on the results of a minimum of eight specimens.

#### 2.2.6. Surface pH of Film

Randomly selected film disks were swollen for 2 h on the surface of an agar plate, prepared by dissolving 2% (wt/vol) agar in warmed simulated saliva (pH 6.8) under stirring and then pouring the solution into a Petri dish until it gelled at room temperature.

Surface pH was measured using a pH meter (HI 2211 pH/ORP Meter, Hanna Instrument, Woonsocket, RI, USA) by placing pH probe in close contact with the wetted patch surface. The surface pH study was carried out by selecting three disks.

#### 2.2.7. Evaluation of the Solubility of FUR Entrapped into Film

In 1 mL of a buffer solution simulating saliva at pH 6.8, at 25 ± 0.1 °C, under stirring, small amounts of free FUR were added until saturation. The suspension was kept under continuous stirring for 1 h, and after the equilibrium was reached, the remaining solid FUR was removed by centrifugation followed by filtration through a 0.45 µm membrane filter (Merk Millipore, Merck S.p.a., Vimodrone (MI), Italy). The clear solution was suitably diluted (1:100) and analyzed by a UV-Vis spectrophotometer at λ_max_ = 331 nm using the appropriate blank and calibration curve (linearity range 0.005–0.1 mg/mL, E_1%salivapH6.8_ = 0.156, corr. coeff. 0.999).

The same experimental protocol was used in order to evaluate the solubility of FUR incorporated into the mucoadhesive film. Three disks, 8.0 mm in diameter, containing 11.5 mg of FUR each, were solubilized in 1 mL of buffer to simulate saliva as previously described. These suspensions were then sonicated at several time intervals, and after the equilibrium was reached, the remaining solid material was removed by filtration through a 0.45 µm membrane filter (Merk Millipore, Merck S.p.a., Vimodrone (MI), Italy). The clear solution was then analyzed. The analyses were conducted in triplicate.

#### 2.2.8. Ex Vivo Mucoadhesion Strength Measurement

To perform the ex vivo mucoadhesive strength evaluation of the prepared film disks, the modified two-armed physical balance method was used [31]. Porcine buccal mucosa excised from just slaughtered pigs was used as model tissue and handled without any pre-treatment. A piece of mucosa was glued with the aid of cyanoacrylate resin (Super Attak Loctite^®^, Henkel Italia Srl, Milan, Italy) on a glass support and kept in a vessel placed in a thermostatic bath at 37 °C ± 1°C. The patch was fixed at the lower side of a rubber stopper with an adhesive and attached to the balance pan. Before starting the measurements, the mucosal tissue was wetted with 50 µL of simulated salivary fluid and then the patch was placed on the tissues so it just touched the mucosal surface and a light force with a fingertip was applied for 20 s. A temperature of 37 °C ± 1 °C was maintained throughout the experiment. The measurements started 5, 10, 15, and 20 min after application, thus allowing for different time contacts. The adhesive strength expressed as the mass (g) required to detach the film from mucosal surface was calculated according to the equation:

Force of adhesion (N) = (g × 9.81)/1000

Then, detachment forces were calculated as:

Detachment force (N/m^2^) = Force of adhesion (N)/Surface area (m^2^)

The maximum adhesive force was determined as the average of the three measurements (*n* = 3).

#### 2.2.9. Swelling and Radial Erosion Tests

A swelling test was conducted by placing a dry film disk (0.5 cm^2^ of area) on a glass support into an analytical balance and weighed. Then, 0.1 mL of artificial saliva at pH 6.8 were added on the disk every 5 min for 50 min. At every time interval, after removal of the excess water with a filter paper, the weight of the wet disk was assessed. The test was performed on one disk of six different batches. Results were reported as means ± SD (*n* = 6; *p* < 0.05).

The swelling degree (SD) was calculated using the following equation:

SD% = (W_s_ − W_d_)/W_d_ × 100

where W_s_ corresponds to the hydrated weight of the sample and W_d_ represents the sample dry weight.

Furthermore, a disk was placed on a glass positioned on graph paper. The experiments were started by placing 0.2 mL of simulated saliva (pH 6.8, 37 °C) on the disk and adding 0.1 mL of saliva every 5 min, for the first 15 min, and then every 15 min. At every time interval, a photograph was taken to evaluate any change in the disk’s morphology.

#### 2.2.10. Drug Release Studies

Drug release in buffer solution simulating saliva was assessed using the flow through system previously described [32]. Briefly, the system consists of a container for the buffer solution simulating saliva (100 mL) from which the liquid is forced into a Plexiglass release chamber. The flow rate of saliva was controlled by a peristaltic pump (Bio-Rad Econo Pump, Hercules, CA, USA) and maintained constant at 0.5 mL/min. In the chamber, the salivary layer wetting the sides of the disk was 0.1 mm thick. The temperature was controlled by submerging the chamber and the container in a thermostatic bath (37 ± 0.1 °C). The drug amount in the solution coming out from the release chamber was then quantitatively determined by UV analysis using the appropriate blank and calibration curve. Results were averaged on six disks from six different batches of film. Every experiment was considered at its end when the complete dissolution of the disk occurred and the amount of drug released matched the original drug content of the disk. Release data were elaborated using Curve Expert v.1.4 and Kaleidagraph v.3.5 software and fitted to the equations applied in release studies from matrix systems. Linear or non-linear least squares fitting methods were used to determine the optimum values for the parameters present in each equation. Fittings were validated by using χ^2^. A *p* value less than 0.05 was considered to be statistically significant.

#### 2.2.11. Permeation Study of FUR Released from the Film through Porcine Sublingual Mucosa

Mucosal specimens consisted of tissue removed from the ventral surface of the tongue of freshly slaughtered domestic pigs. Specimens were prepared as described previously [17]. Briefly, specimens, used within 2 h from animal sacrifice, were dipped for approximately 1 min in saline solution previously warmed to 60 °C; the connective tissue was then carefully peeled off from the mucosa (slides 150 ± 25 µm thick) to obtain the heat-separated epithelium along with the intact basal lamina. The thickness was measured using a digital micrometer. Before the beginning of the experiments, specimens were equilibrated in phosphate buffered saline pH 7.4 (PBS) for about 3 h at room temperature to remove biological matter which could interfere with drug analyses. The equilibration medium was replaced with fresh PBS every 15 min.

Appropriate sections of mucosa were mounted in vertical jacketed, Franz type diffusion cells (Permeagear, flat flange joint, 9 mm orifice diameter, 15 mL acceptor volume, SES GmbH—Analysesysteme, Bechenheim, Germany) used as a two compartment open model. Tissue disks (12 mm diameter) were equilibrated for 1 h at 37 ± 0.1 °C adding PBS in both the donor and the acceptor compartment. This step was followed by the removal of PBS from the donor compartment and replacement with one patch (8 mm of diameter) in 0.4 mL of simulated saliva applied to the apical side of the membrane. At regular time intervals (30 min), samples (0.5 mL) were withdrawn from the acceptor compartment and the sample volume was taken out and replaced with fresh fluid. Each experiment was carried out at 37 ± 0.1 °C for 6 h and repeated six times using different fractions of the same portion of tissue. The permeated FUR was determined by UV analysis using the appropriate blank and calibration curve (linearity range 0.005–0.1 mg/mL, E_1%PBSpH7.4_ = 0.152, corr. coeff. 0.999). At testing concentrations, PBS, artificial saliva, and formulation components do not interfere with FUR quantification.

Drug flux (*Js*) through the mucosal membrane was calculated at the steady state per unit area by linear regression analysis of permeation data following the relationship *Js* = *Q*/A*t* (mg/cm^2^ h), where *Q* is the amount of the drug which passes through the cell layers into the acceptor compartment, A is the active cross-sectional area available for diffusion (0.636 cm^2^), and *t* is the time of exposure (h).

The permeability coefficient (*Kp*) was then calculated according to the equation *Kp* = *Js*/*Cd* (cm/h), where *Cd* is the drug concentration in the donor compartment (mg cm^−3^).

The analyses were performed on two disks of six different batches. Results were reported as means ± SD (*n* = 12; *p* < 0.05).

## 3 Results and Discussion

### 3.1. Film Formulation

Because of low costs and ease of production, one of the most common approaches to get delivery systems with suitable drug release is to embed the drug in an adequate matrix. The film as matrix system is also considered the most suitable in order to obtain a solid dispersion of drugs in their amorphous form. In particular, formulation of a bioerodible mucoadhesive film in which FUR was homogenously dispersed has been designed with the objective of improving drug bioavailability by an adequate delivery throughout the mucosa.

Therefore, a readily water-soluble polymer such as Eudragit^®^ L-100 was chosen for the formulation as the matrixing polymer, together with PVP-K90 for conferring mucoadhesive properties. Eudragit^®^ L-100 (Methacrylic Acid Copolymer Type A USP/NF, Methacrylic Acid—Methyl Methacrylate Copolymer 1:1 Ph. Eur.) is a biocompatible, mucoadhesive anionic polymer. Its solubility is closely influenced by pH, and specifically, increases for pH values above 6. This polymer, when dispersed in the aqueous mixture containing all the formulation components, confers on the mixture a low viscosity, which is useful for pouring and homogeneously distributing it into the mold, without loss of product. When dried, Eudragit^®^ L-100 is able to maintain FUR in its amorphous form and is useful for preparing drug loaded films that ensure a fast release of the drug.

The characteristics of flexibility and softness of the films have been conferred by the introduction in the formulation of wetting agents and plasticizers [33]; in particular, Sorbitol and Propylene Glycol are chosen for their ability to retain water within the film and for their inhibitory activity on bacterial growth especially when they are in association.

The quantitative composition of films has been selected on the basis of our previous experiences [34]. Films with different matrix component ratios have been tested to select the composition with the highest FUR loading capacity and adequate characteristics of mucoadhesion and drug discharge.

Mucoadhesive sublingual films of FUR have been prepared by the solvent casting method. The method is simple, inexpensive, and does not imply the use of organic solvents. The method has been the most appropriate to obtain a solid dispersion of drug in a matrix system with a high reproducibility of results.

### 3.2. Film Weight, Thickness, and Drug Load Uniformity

The reproducibility of the films preparation has been assessed by measuring the average weight (48.50 ± 1.46 mg), thickness (0.750 ± 0.022 mm), drug content (11.53 ± 0.43 mg), and surface pH (7.2–7.4) of disks (area 0.5 cm^2^) from different batches. All data are in accordance with the requirements of the Italian Pharmacopoeia [FU XII ed] and confirm high product reproducibility.

### 3.3. Differential Scanning Calorimetry Analysis

**Figure 1:**
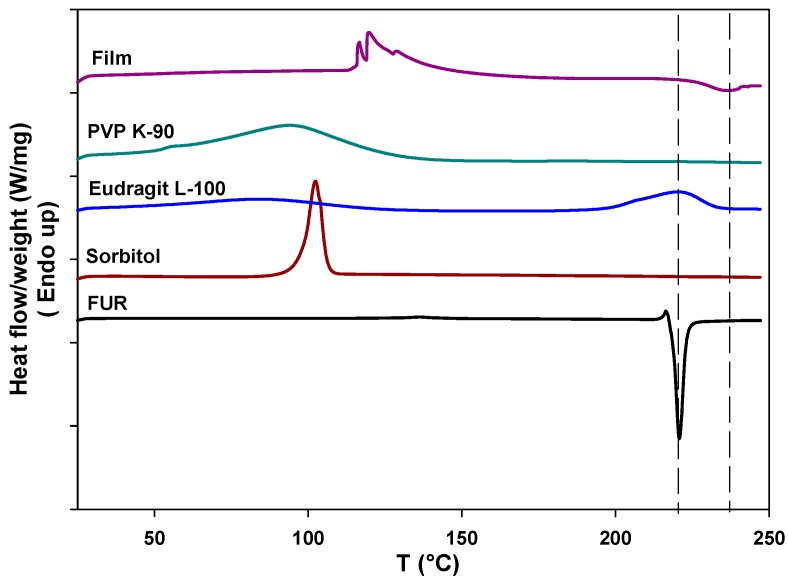
Differential Scanning Calorimetry (DSC) thermograms of the Furosemide (FUR)-loaded film and of all the individual components present in the film.

Drug-polymer interactions were investigated using differential scanning calorimetry (DSC). Thermograms of the single components of the film and their mixtures in the film have been recorded. FUR thermograms show a small peak at 219 °C characteristic of the onset of drug melting. This peak is followed by a sharp exothermic peak at 220 °C attributable to drug decomposition that takes place upon melting [35]. The thermogram of sorbitol shows a typical sharp melting peak at about 100 °C, while Eudragit presents two endothermic peaks, at 66 and 220 °C, attributable to unbound water and to the condensation in anhydride, respectively. PVP is an amorphous polymer and the only feature in its thermogram is associated with the evaporation of absorbed water. The thermogram of the FUR-loaded film (named “Film” in Figure 1) shows a group of endothermic peaks that can be attributed to the melting of sorbitol domains and a shallow and broad endothermic peak remnant of FUR degradation. This last evidence supports the theory that the drug is embedded within the polymer matrix mostly in the amorphous form. The disappearance of Eudragit peaks, when it is in the form of the film together with the other components, suggests that the film forming process has removed the water, and condensation reactions are impeded or shifted to higher temperatures.

### 3.4. Attenuated Total Reflectance (ATR)

**Figure 2:**
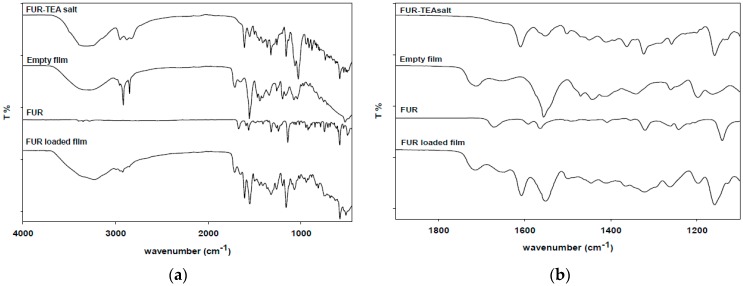
(**a**) Attenuated Total Reflectance-Fourier Transform Infrared Spectrometer (ATR-FTIR) full spectra of FUR-loaded film, empty film, FUR, and FUR-Triethanolamine (FUR-TEA) salt; (**b**) Magnification of the 2000–1000 cm^−1^ portion of the spectra.

The formation of FUR-TEA salt was verified through ATR-FTIR spectroscopy. Spectra of FUR, FUR-TEA salt, unloaded film, and film loaded with FUR are shown in Figure 2a,b. In the spectra of FUR-TEA and film loaded with FUR, a sharp peak at 1610 cm^−1^, associated with the carbonyl stretching of the carboxylate anion (–COO^−^), is present (this peak is absent in the unloaded film) together with a shift from 1141 cm^−1^ to 1159 cm^−1^ due to the stretching of the C–N band, indicating that FUR is entrapped in the film as triethanolamine salt [36].

### 3.5. Mechanical Tests

Young’s modulus (E), yield strength (YS), and elongation at break (EB) are parameters that provide an indication of the strength and elasticity of the patch and characterize the mechanical performance of the film. These parameters are useful to evaluate the manageability of the film, which has to be handled by patients without breaking. It is suggested that a suitable buccal patch should have a relatively high YS and EB [37,38]. The measured Young’s Modulus was 70.22 ± 7.7 MPa, the yield strength was 5.3 ± 0.55 MPa, and the relative EB% was 259 ± 0.85%. The drug-loaded film can be classified as an elastomer [39].

### 3.6. Surface pH

The surface pH of the films ranged from 6.71 to 6.93. Since the detected pH value is compatible with the oral cavity, there will not be any kind of irritation to the mucosal tissue.

### 3.7. Solubility Test

The assessment of solubility in simulated saliva at pH 6.8 showed that FUR dissolves to a maximum of 3.18 mg/mL. When the FUR was incorporated in the matrix film its solubility significantly increased up to 28.36 mg/mL, approximately nine-fold higher than the value obtained for the free FUR.

### 3.8. Ex Vivo Mucoadhesion Strength Measurement

The mucoadhesive properties of sublingual film are of great importance as they influence the ability of the dosage form to be retained at the site of action, in intimate contact with the absorption membrane. Ex vivo mucoadhesion strength measurements were conducted with a modified two-armed physical balance. It also evaluated the variation in adhesive strength as a function of contact time.

The obtained results (Table 1) are expressed as force of adhesion and detachment force, whose equations were previously described.

**Figure 3:**
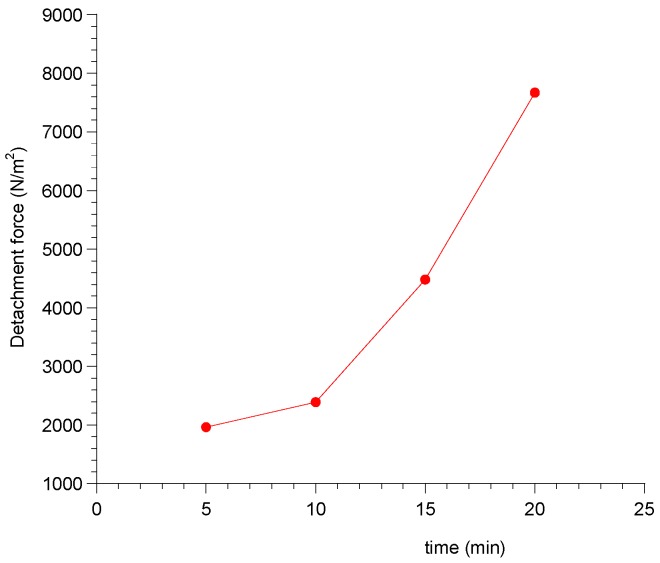
Exponential trend of detachment force of the patch as a function of contact time on porcine buccal mucosa.

The mucoadhesive force grew with increasing contact time of the patch and the hydration of the latter. In particular, by increasing the time contact between the patch and mucosa, an exponential increase of the forces required to detach the patch were observed (Figure 3).

### 3.9. Swelling and Radial Erosion Tests

**Figure 4:**
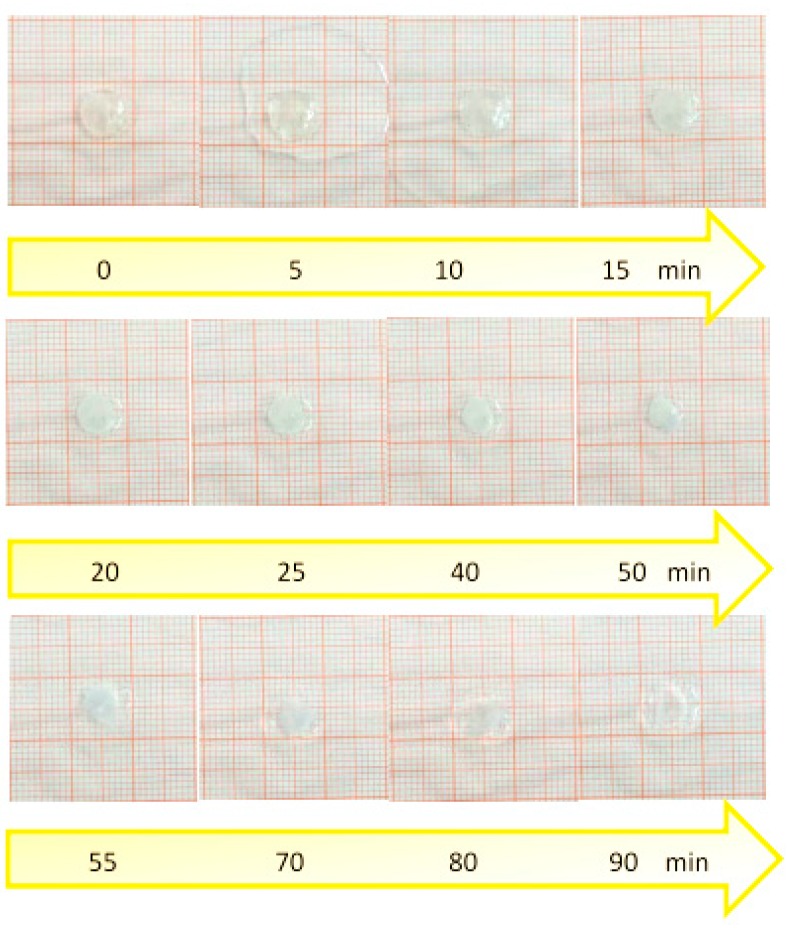
Timeline from 0 to 90 min of the radial swelling of the patch.

The radial erosion test was carried out in order to measure the surface area of the sublingual cavity which is effectively covered by the film when dissolution occurs. The radial erosion test showed that, after an initial swelling of the film that occurs in the first 10 min due to the absorption of saliva, the disk undergoes rapid erosion and loss of weight until the total dissolution, in about 90 min (Figure 4).

**Figure 5:**
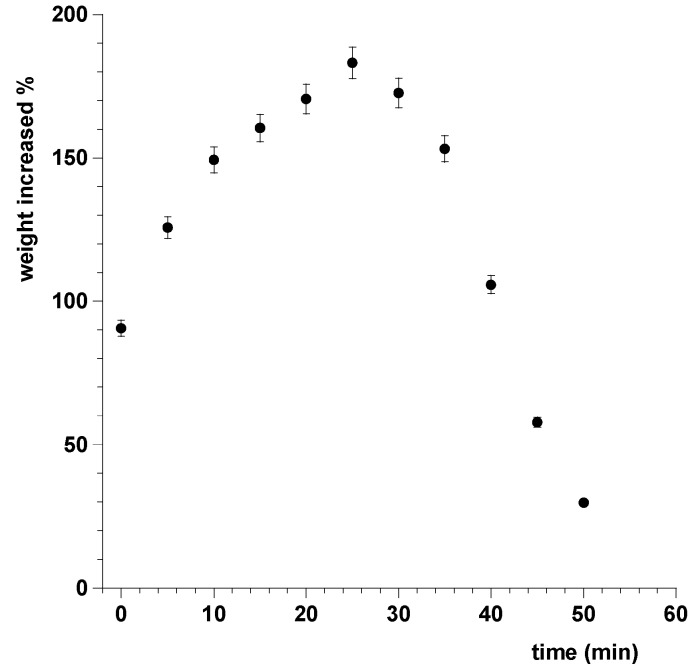
Swelling index measured as percent of weight increased vs. time. Values are presented as means ± SD (*n* = 6).

The data related to the weight variation showed that the weight of the disk increases in the first 25 min-interval due to the absorption of saliva and the consequent swelling. After that time, however, the weight decreases due to the dissolution of the dosage form and the removal of the solution of the drug (Figure 5).

These data indicate that the film absorbs the saliva which is useful for the drug dissolution, but the swelling of the film is not so great as to cause discomfort for the patient.

### 3.10. Drug Release Studies

**Figure 6:**
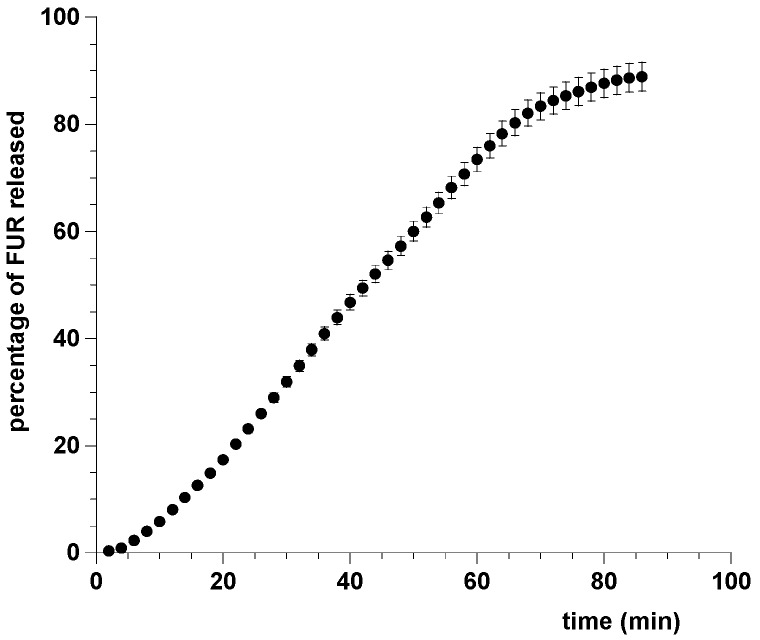
Cumulative percent of FUR released from patch in simulated saliva pH 6.8. Values are presented as means ± SD (*n* = 6).

Usually, drug release from tablets is studied according to the official pharmacopoeias. Current test methods require large volumes of dissolution medium and are operated under sink or pseudo-sink conditions. These methods do not simulate the conditions prevailing in buccal environment where small amounts of liquid exist; non-sink conditions are more appropriate for describing the behavior of medications. Indeed, for buccal dosage forms, an initial fast release cannot be measured with the existing official methods, and in vitro dissolution tests should be performed in small volumes of dissolution medium. For these reasons, release tests were performed using a flow through cell system able to simulate the sublingual conditions, in particular, the saliva turnover in the oral environment [34,40]. This study, assessing the drug release from the dosage form when exposed to small volumes of fresh salivary fluid that is continuously replaced, showed that FUR is discharged from film quickly; in particular, about 80% of FUR was released from the dosage form within 60 min. Experimental results were reported in Figure 6.

To understand the mechanism of drug discharge, the most common models (zero order, exponential, Higuchi, power law, and Weibull) used in dissolution analysis were curve fitted to our experimental data, expressed as mean of dose fraction released versus time (Table 2).

The best fit was obtained using the Weibull function (Equation (1)), characterized by an S-shaped trend with upward curvature followed by a flattening [41].

(1) $$\frac{M_{t}}{M_{\infty }}=1-exp^{\left(-at^{b}\right)}$$

where *a* and *b* are constants.

By fitting to the entire set of data with the Equation (1), the *a* and *b* parameters resulted in 9.8 × 10^−4^ ± 0.56 × 10^−4^ and 1.75 ± 0.0146, respectively. The best fit was confirmed by *R* value (0.99955), standard error (0.00931), χ^2^ value (0.003552), and analysis of residuals.

This behavior indicated that a complex mechanism governs the dissolution process, highlighted by *b* higher than 1. The drug diffusion through the matrix network depends on the polymer/drug’s physical and chemical characteristics. Probably, the relatively low solubility of FUR plays a role in the dissolution process and in the release kinetics along with other release mechanisms [42].

### 3.11. Permeation Study of FUR Released from the Film through Porcine Sublingual Mucosa

The ability of FUR discharged from the matrix film to cross the sublingual mucosa and reach the systemic circulation has been analyzed by performing ex vivo permeation studies using vertical Franz type diffusion cells and porcine sublingual mucosa as the most useful model to simulate human epithelium [43].

**Figure 7:**
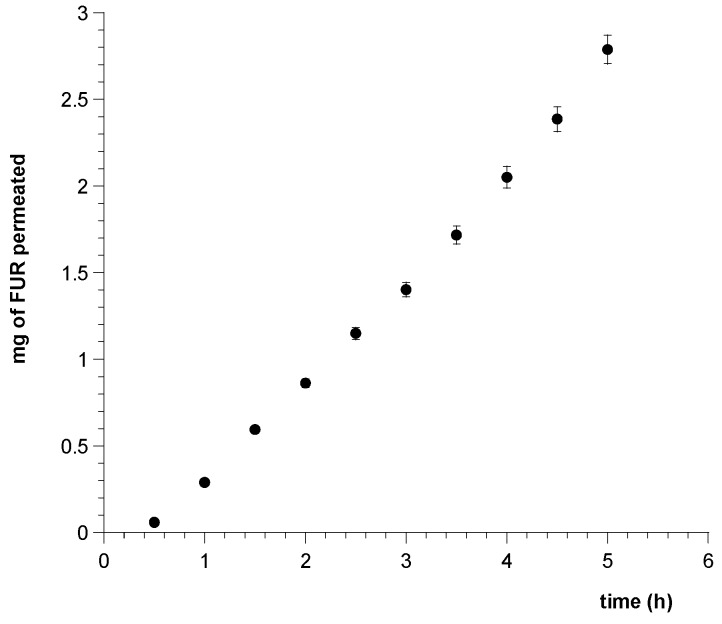
Plot of cumulative amount of FUR permeated across porcine sublingual mucosa vs. time from a patch (0.5 cm^2^) soaked with simulated saliva. Values are presented as means ± SD (*n* = 12).

Figure 7 shows drug movement from the patch to the serosal side of porcine tissue, expressed as cumulative amount of permeated FUR versus time.

Extrapolating the flux (*Js*) per unit area of FUR through the mucosal membrane at the steady state showed that the new dosage form applied on porcine sublingual mucosa for six hours produced a massive input of FUR in the acceptor compartment with a drug flux (*Js*) of 1.2211 ± 0.1115 mg/cm^2^ h (*R* = 0.9981) and permeability coefficient (*Kp*) of 0.05309 ± 0.00485 cm/h.

Considering that in our experiments the drug release occurred from one side of the patch adhered to the mucosa and in in vivo conditions each patch is in contact with both the floor of the mouth and the ventral surface of the tongue, a patch of 1 cm^2^ produces a drug flux of about 2.5 mg/cm^2^ h.

A sublingual drug delivery system with optimal performance should release drug amounts adequate to reach effective plasma levels. These levels can be achieved when the rate of drug entry in the systemic circulation is equal or greater than the rate of drug disappearance from the blood. Drug declining follows a first order kinetic equation and is equal to the product of the plasma concentration (*Cp*) with the first order rate constant of elimination (*K*_e_). In turn, *K*_e_ can be calculated by the biological half-life of the drug. In other words:

Drug input rate = Drug output rate = *Cp* × *K*_e_

where *K*_e_ = 0.693/*t*_1/2_.

Taking into account the pharmacokinetic parameters of FUR, after oral administration of 20 mg of FUR tablets [29] (*t*_1/2_ about 1.9 h; volume of distribution *V*_d_ about 14 L; C_max_ = 552 ng/mL), the rate of FUR elimination was calculated (*V*e = Cp × Ke) as 201 µg·L·h^−1^ and the amount of FUR that should reach the systemic circulation to obtain drug levels comparable to *per os* administration was predicted as 2.8 mg/h (Vd × Ve) Therefore, on the basis of pharmacokinetics literature data, it is possible to hypothesize that by administration of a 1 cm^2^ patch therapeutic plasma levels of FUR can be achieved.

Furthermore, due to both the drug release results and radial swelling tests, we can highlight that within 60 min the dosage form dissolved, leaving a highly concentrated drug solution that can be spread over a wider surface of the floor of the mouth, increasing the surface area available for absorption and, consequently, reducing the time to achieve therapeutic concentration.

The calculated plasma levels of the drug, reached by means of the designed drug delivery system, indicate the patch’s suitability for use in the management of hypertension. Nevertheless, the in vitro approach for new dosage forms has inherent weaknesses since it cannot capture all the differences that the in vivo environment can offer, being affected by several parameters, i.e., salivary turnover, pH, health state of mucosa, and the drug’s enzymatic metabolism in the tissue. Additional work would be needed to understand the possible implications in pharmacokinetics and our assumptions should be verified in vivo on animal models.

## 4. Conclusions

This study aimed at the development and evaluation of a new solid dosage form, a sublingual patch, in order to allow a massive release of FUR through the sublingual mucosa for hypertensive management. A comprehensive set of characterizations have been performed showing that the formulated mucoadhesive patch is flexible and resilient, although thinner than most patches produced for the purpose; its weight and drug content uniformity meet the Pharmacopoeia criteria. Moreover, it shows good mucoadhesive properties and a high dissolution and drug release rate.

In particular, ex vivo studies have shown that the permeation of FUR released from film through the sublingual mucosa occurs at a rate that is suitable to ensure the achievement of therapeutic concentrations in the bloodstream. All the results gathered here suggest that the prepared matrix film possesses adequate physio-chemical and drug release properties. Used as sublingual film, it can enhance drug solubility and bioavailability, thus providing a better drug utilization and determining a rapid onset of action. In addition to being able to circumvent the pitfalls of conventionally administered systems, the proposed patch boasts a high level of patient acceptance.

## Figures and Tables

**Table 1:** Force of adhesion and detachment force of the patch after different contact time on porcine buccal mucosa (*n* = 3).

Contact Time (min)	Force of Adhesion (N)	Detachment Force (N/m^2^)
5	0.098	1960
10	0.1196	2392
15	0.2242	4485
20	0.3834	7667

**Table 2:** Mathematical models fitted to experimental release curve.

Model	Equation	Correlation Coefficient	Standard Error
Zero order *	$Q_{t}=Q_{0}+K_{0}t$	0.99714	0.01565
First order *	$Q_{t}=Q_{0}\;exp^{-Kt}$	0.97091	0.04961
Higuchi *	$\frac{M_{t}}{M_{\infty }}=k\sqrt{t}$	0.84868	0.10737
Korsmeyer–Peppas *	$\frac{M_{t}}{M_{\infty }}=at^{n}$	0.99864	0.01078
Weibull **	$\frac{M_{t}}{M_{\infty }}=1-exp^{\left(-at^{b}\right)}$	0.99955	0.00931

* fitted with the first 60% of the release data; ** fitted with the entire set of data
